# IL-10 and TGF-β1 gene polymorphisms in Greek patients with recurrent aphthous stomatitis

**DOI:** 10.4317/medoral.25352

**Published:** 2022-06-05

**Authors:** Vasileios Kounoupis, Dimitrios Andreadis, Maria Georgaki, Eleni Albanidou-Farmaki, Michail Daniilidis, Anastasios Markopoulos, Nikolaos Karyotis, Nikolaos G Nikitakis, Athanasios Poulopoulos

**Affiliations:** 1 PhD, Department of Oral Medicine/Pathology, School of Dentistry, Aristotle University of Thessaloniki, Greece; 2 Associate Professor, Department of Oral Medicine/Pathology, School of Dentistry, Aristotle University of Thessaloniki, Greece; 3 PhD, Department of Oral Medicine and Pathology and Hospital Dentistry, School of Dentistry, National and Kapodistrian University of Athens, Greece; 4 Associate Professor, Department of Oral Medicine/Pathology, School of Dentistry, Aristotle University of Thessaloniki, Greece; 5 Emeritus Professor, 1st Department of Internal Medicine, School of Medicine, AHEPA Hospital, Aristotle University of Thessaloniki, Greece; 6 Professor, Department of Oral Medicine/Pathology, School of Dentistry, Aristotle University of Thessaloniki, Greece; 7 Biologist, Immunogenetics Laboratory, 1st Department of Internal Medicine, School of Medicine, Aristotle University of Thessaloniki, Greece; 8 Professor and Chair, Department of Oral Medicine and Pathology and Hospital Dentistry, School of Dentistry, National and Kapodistrian University of Athens, Greece; 9 Professor and Chair, Department of Oral Medicine/Pathology, School of Dentistry, Aristotle University of Thessaloniki, Greece

## Abstract

**Background:**

Recurrent aphthous stomatitis (RAS) is one of the most frequent inflammatory disorders of the oral mucosa. Cytokines, which play an important role in RAS pathogenesis, participate directly or indirectly in normal, immunological and inflammatory processes and are secreted from cells belonging to innate and adaptive immunity as a consequence of microbial and antigenic stimuli. Gene polymorphisms in specific cytokines may predispose to RAS development. The aim of this study was the investigation and association of IL-10 and TGF-β1 gene polymorphisms with RAS.

**Material and Methods:**

Study’s cohort consisted of 60 Greek patients diagnosed with RAS, including 40 patients with minor, 10 patients with major and 10 with herpetiform aphthous ulcers. Forty age- and sex-matched control subjects were included in this study. DNA was extracted from whole blood samples of all patients and sequence-specific primers (SSP)-based polymerase chain reaction (PCR) was used for genotyping. Gene polymorphisms for cytokines IL-10 at loci -592 and -819 and for TGF-β1 at codon 10 were detected.

**Results:**

Significant differences between patients with minor RAS and healthy controls were recorded for IL-10 genotypes distribution at position -592 (*p*=0.042) and -819 (*p*=0.045) with predominance of C/A and C/T genotypes in RAS patients, respectively. Also, in patients with minor and herpetiform aphthous ulcerations, heterozygous TGF-β1 genotype C/T at codon 10 was associated with increased risk of RAS (*p*=0.044 and *p*=0.020, respectively).

**Conclusions:**

These data provide evidence that genetic predisposition for RAS and possibly its specific clinical variants is related with the presence of gene polymorphisms for specific cytokines, including IL-10 and TGF-β1, which, in turn, may vary according to geographic origin and genetic background.

** Key words:**Recurrent aphthous stomatitis, aphthae, IL-10, TGF-β, gene polymorphisms, oral mucosa.

## Introduction

Recurrent aphthous stomatitis (RAS) belongs to the group of chronic inflammatory, ulcerative diseases of the oral mucosa, characterized by recurrent, solitary or multiple ulcers (1). Three clinical variations of RAS are recognized: a) Minor, affecting about 80% of patients with RAS; ulcers are usually less than 5 mm in diameter, and usually occur on the labial or buccal mucosa and the ﬂoor of mouth, while involvement of the keratinized mucosa is uncommon; the ulcers heal within 7-14 days without scarring (2). b) Major, a less common, severe form of RAS with larger ulcerations, which can occur anywhere in the mouth, but most commonly involve the lips, soft palate and tonsillar fauces, persisting from 3 to 6 weeks; they usually have their onset after puberty and are chronic, persisting for up to 20 or more years, and often heal with scarring, which over time may become significant, even leading to restricted mouth opening. c) Herpetiform aphthous ulceration (HAU), the least common variety with a predisposition for women and a later age of onset; it is characterized by recurrent crops of multiple, as many as 100, widespread small, painful ulcers, each measuring 2-3 mm in diameter, that tend to coalesce producing large irregular ulcers (1-2).

According to the prevailing theory the etiopathogenesis of RAS is associated with the activation of the inflammatory and immune host response against various antigenic stimuli (3). No single triggering agent is responsible and genetic predisposition, immunological factors, trauma, hormonal influences, hematologic abnormalities, systemic disorders, stress, infectious agents, allergies, nutritional deficiencies and drugs are considered as factors participating in RAS etiopathogenesis (1,3,4). There is recent evidence that genetically-mediated disturbances of the innate and acquired immunity play an important role in RAS development (5).

The pathogenetic mechanism leading to mucosal destruction in RAS appears to represent a T-cell mediated immunologic reaction (6). Analysis of the peripheral blood in patients with recurrent aphthous ulcerations shows a decreased ratio of CD4+ to CD8+ T lymphocytes, increased T-cell receptor γδ+ cells and increased TNF-α generated by T-cells, macrophages and mast cells (7). In addition, aphthous lesions exhibit a mononuclear cell infiltrate with a predominance of T-helper cells before epithelial breakdown (8); deficiencies in TLR activity may also affect the T-helper cell response in patients with RAS (9). Cytotoxic-suppresor T cells are predominant in the subsequent ulcerative phase, to be later replaced by helper cells during healing (5). It has been shown that these histopathologic processes are initiated by the increased production of cytokines by activated macrophages, T-lymphocytes and mast cells as a consequence of microbial and antigenic stimuli (4,10). In RAS patients, several dysregulations in cytokine expression have been recorded, including elevated levels of IL-2 and lower levels of IL-10 in lesional mucosa (11).

In 2001, six immune mediators (IL-10, IL-19, IL-20, IL-22, IL-24 and IL-26) were grouped into the so-called IL-10 family of cytokines. The genes encoding human IL-10, IL-19, IL-20, and IL-24 are located on the chromosome 1 (1q32). The human IL22 and IL26 genes are located on the longer arm of chromosome 12, on 12q15 (12,13). The main source of IL-10 *in vivo* appears to be monocytes, macrophages, T-cell subsets, dendritic cells, B-cells, NK-cells, mast cells, as well as neutrophilic and eosinophilic granulocytes. The function of activated macrophages and dendritic cells can be arrested by IL-10, and this cytokine controls innate and cellular immune response (12). The pleiotropic activities of IL-10 family members are mediated by specific cell surface receptor complexes that each consist of an R1 and R2 chain, both being members of the CRF2, and signaling induced by cytokine binding to CRF2 members is known to occur primarily via the JAK/STAT pathway. IL-10 suspends production of IL-12 and expression of co-stimulator and MHC class II molecules from macrophages and dendritic cells (12,14). IL-10 intervenes in healing stage of the disease by stimulating epithelial cell proliferation. Consequently, epithelialization could be delayed in RAS patients with low serum IL-10 levels, prolonging the duration of the disease (8,9).

The cytokine TGF-β is a pleiotropic growth factor with important anti-inflammatory and immunosuppresive properties. It is noteworthy to mention that TGF-β suppresses production and proliferation of activated T-cells by exerting inhibitory effects on IL-1 and IL-2 production (15). TGF-β1 is the most frequent isoform in various tissues and is structurally a 25-kDa homodimer protein which consists of two 12.5-kDa subunits with one disulfide bond (15).

IL-10 and TGF-β anti-inflammatory cytokine production was found to be decreased in RAS patients compared to healthy individuals (16). These results indicate that disproportion in cytokine production may be a contributing factor in the pathogenesis of RAS. Moreover, imbalance in pro- and anti-inflammatory cytokine network may lead to the breakdown of peripheral tolerance in RAS and the excessive immune response towards harmless micro-organisms colonizing oral mucosa or self-antigens (16).

The aim of this study is to investigate cytokines IL-10 and TGF-β1 gene polymorphisms and haplotypes frequencies in Greek patients with RAS in comparison with healthy subjects. Our main hypothesis is that cytokines IL-10 and TGF-β1 gene polymorphisms in specific locations are associated with an increased risk of developing RAS and are possibly related with the prevalence of specific clinical variants (minor, major, or herpetiform ulcers).

## Material and Methods

- Patients

IL-10 and TGF-β gene polymorphisms were examined in a cohort study of 60 Greek patients with RAS manifestations, who were consecutively examined and diagnosed in the Department of Oral Medicine/Pathology in Aristotle University of Thessaloniki. Forty patients were affected by minor aphthous ulcers, 10 patients demonstrated major aphthous ulcers and 10 presented with herpetiform ulcers. Forty age- and gender-matched healthy control subjects were included in this study. Sample size was determined based on power analysis calculations. The demographic and clinical characteristics of each group (RAS patients and control healthy subjects) are summarized in Table 1.

**Table 1:**
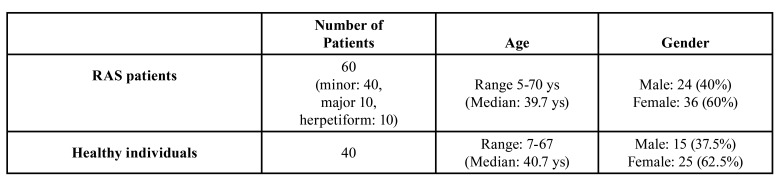
Summary of demographics of RAS patients and healthy control subjects.

Exclusion criteria included autoimmune diseases (such as erythema multiforme, Stevens-Johnson syndrome, lichen planus, lupus erythematosus, pemphigus, mucous membrane pemphigoid, and Sjögren syndrome), gastrointestinal disorders (such as Crohn’s disease, idiopathic inflammatory bowel disease), granulomatous diseases (e.g. sarcoidosis, orofacial granulomatosis), anemia (iron-deficiency anemia, B12 and folic acid deficiencies), cyclic neutropenia, Adamantiades-Behçet's syndrome, autoinflammatory disorders (PFAPA syndrome, HIDS and TRAPS syndromes), diabetes mellitus, Sweet syndrome, Reiter syndrome, HIV and Helicobacter pylori-infected patients were excluded from our study. The diagnosis of HP infection was based on the detection of speciﬁc immunoglobulin G (IgG) and immunoglubulin A (IgA) antibodies in the serum of the patients. Another reliable non-invasive diagnostic tests for H. pylori detection were INFAI 13C-Urea Breath Test and Helicobacter Pylori Stool detection. This study protocol was approved by the Ethical Committee of School of Dentistry of Aristotle University of Thessaloniki (reference number: 39/16-05-2018); all participants provided informed consent.

- Sample collection and DNA extraction

Five ml of whole blood was extracted from all patients with recurrent aphthae, as well as of all control subjects, and the material was collected in a 10 ml vial with EDTA which was used as anticoagulant.

DNA extraction followed standard molecular laboratory procedures, the basic steps of which involved the following: First, 500 μl of whole blood or buffy coat were placed in a 2 ml polypropylene microcentrifuge tube, adding 1 ml of cold (0-50C) Red Cell Lysis Buffer. Subsequently, 125 μl of Protease in Nuclear Lysis Buffer (4mg/ml) were added and the sample was placed in a 650C heat block or water bath for 10 minutes. In turn, 275 μl of Protein Clearing Solution were added and the supernatant (containing DNA) was transferred to a clean, labeled 2.0 ml tube. Then, 500 μl of ice cold (0-50 C) 90% ethanol were sypplemented and, finally, 150 μl of DNA Suspension Buffer or sterile distilled water were added to the tube.

- Genotyping

In this study, sequence-specific primers (SSP)-based polymerase chain reaction (PCR) was used for genotyping and, after PCR amplification, the amplified DNA fragments were segregated in agarose gel according to their electrophoretic capability. The Invitrogen™ Cytokine Genotyping Kit (Invitrogen, Carlsbad, CA, USA) consisted of various formulations of lyophilized primer mixes that were used to amplify genomic DNA using a 96-well thermal tray.

A mixture of a reaction buffer with a human genomic DNA sample and Tag DNA Polymerase was used for initiation of the Genotyping Kit. The following steps were the dispensation of the mixture to a 96-well tray, coverage of the tray, and thermal cycling. After thermal cycling, the product was loaded onto a 2% agarose gel for electrophoresis. The electrophoretic band product with the addition of ethidium-bromide dye became visible following exposure to UV light and was then detected by photographic film using a worksheet for specific amplifications patterns.

- Statistical analysis

Statistical signiﬁcance of differences between RAS cases and control group distributions for alleles and genotypes was determined using SNPStats, which can analyze data from association studies based on SNPs or biallelic markers. The software main page can be found online http://bioinfo.iconcologia.net/SNPstats. The software is used following three steps, namely data entry, data processing and analysis customization, maintaining the possibility of performing multiple analyses in one session. Anonymous use was guaranteed and data were treated as confidential. A signiﬁcance level of *P*< 0.05 was used.

The analysis of association for each SNP was performed both for quantitative or binary response variables. For binary responses, the logistic regression analysis was summarized with genotype frequencies, proportions, odds ratios (OR) and 95% confidence intervals (CI). For quantitative responses, linear regression was summarized by means, standard errors, mean differences with respect to a reference category and 95% CI of the differences (17).

## Results

The demographic and clinical characteristics

All samples (from RAS patients and control subjects) were processed with usage of Invitrogen Cytokine Genotyping Kit and gene polymorphisms were detected for cytokines IL-10 at loci -592 and -819 and for TGF-β1 at codon 10.

- IL-10

According to our investigation, gene polymorphisms for IL-10 at loci -592 and -819 were associated with increased risk of RAS in patients with minor recurrent aphthae, but not in patients with major or herpetiform aphthae (Table 2).

**Figure 1:**
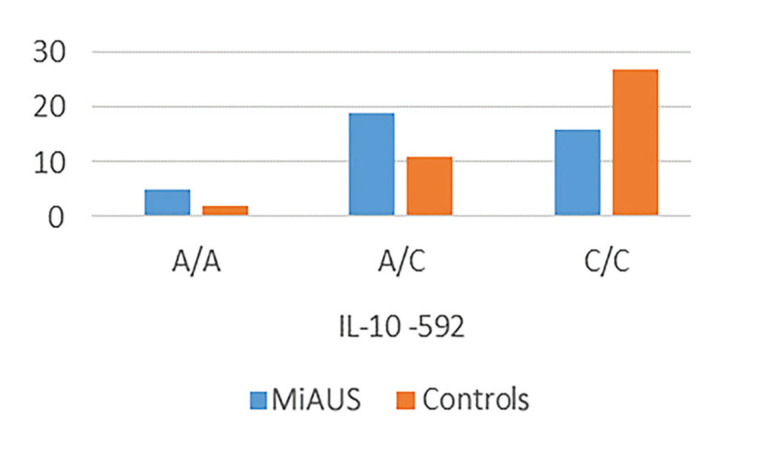
Differences in the expression of IL-10 gene polymorphisms/haplotypes at location -592 between patients with minor aphthous ulcerations (MiAUS) and healthy control subjects.

Specifically, in patients with minor recurrent aphthous ulceration manifestations, significant differences in allele frequencies and genotypes distribution at position -592 were recorded. Percentages of genotypes A/A, C/A, C/C were 12%, 48% and 40% in patients’s group, versus 5%, 28% και 68% in control group, respectively (*p*=0.042) (Table 3 and Fig. 1).

**Table 2:**
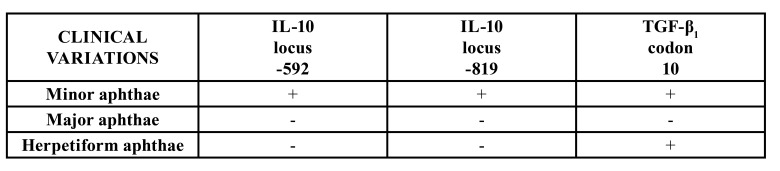
Statistically significant differences in genotype distribution of studied cytokines between clinical variations of the disease and healthy control subjects.

**Table 3:**
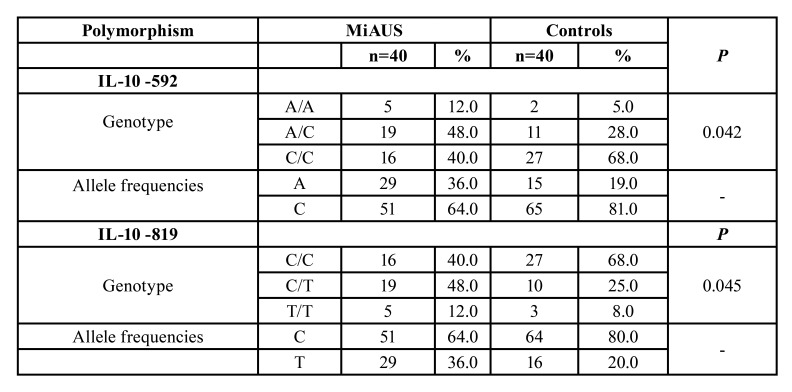
Distribution of the IL-10 genotype polymorphisms at locations -592 and -819 in patients with minor aphthous ulcerations (MiAUS) and healthy control subjects.

**Figure 2:**
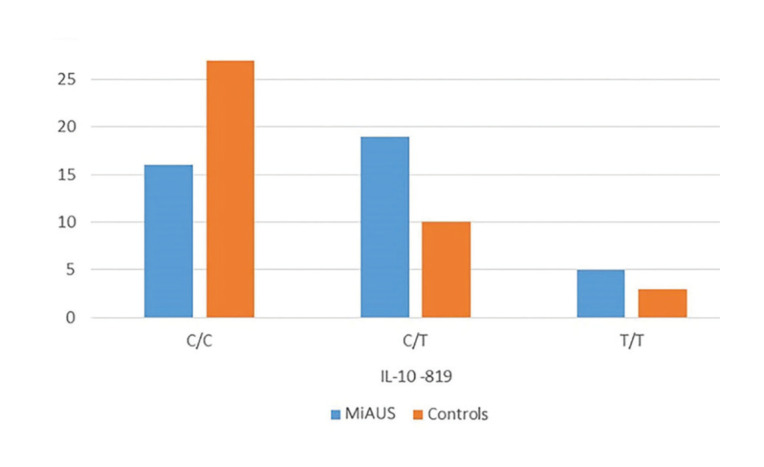
Differences in the expression of IL-10 gene polymorphisms/haplotypes at location -819 between patients with minor aphthous ulcerations (MiAUS) and healthy control subjects.

Moreover, in minor RAS patients’ group, significant difference in IL-10 genotypes distribution at position -819 was recorded (CC: 40%, CT: 48%, TT: 12%), as compared to controls (CC: 68%, CT: 25%, TT: 8%) (*p*=0.045) (Table 3 and Fig. 2).

- TGF-β1

According to our investigation, gene polymorphisms for TGF-β1 at codon 10, specifically heterozygous genotype C/T, were associated with increased risk of RAS manifestation in patients with minor recurrent and herpetiform aphthae, but not in patients with major aphthae (Table 2).

**Figure 3:**
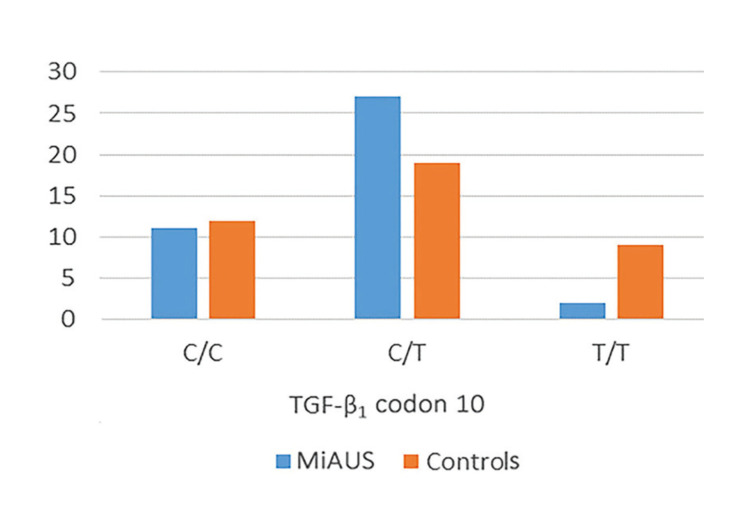
Differences in the expression of the TGF-b1 gene polymorphisms/haplotypes at codon 10 between patients with minor aphthous ulcerations (MiAUS) and healthy control subjects

Specifically, in patients with minor RAS, an increased C/T genotype frequency (68%) was noticed in comparison to controls (48%). On the other hand, C/C and T/T homozygous genotypes accounted for 28% and 5%, respectively, in patients’ group, while the corresponding percentages in controls were 30% and 22% (*p*=0.044) (Table 4 and Fig. 3).

**Table 4:**
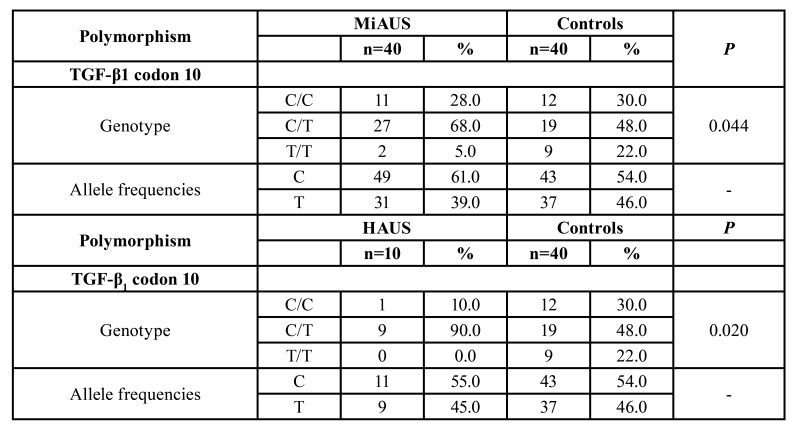
Distribution of the TGF-β1 genotype polymorphisms at codon 10 in RAS patients, with minor aphthous ulcerations (MiAUS) and herpetiform aphthous ulcerations (HAUS), and healthy control subjects.

**Figure 4:**
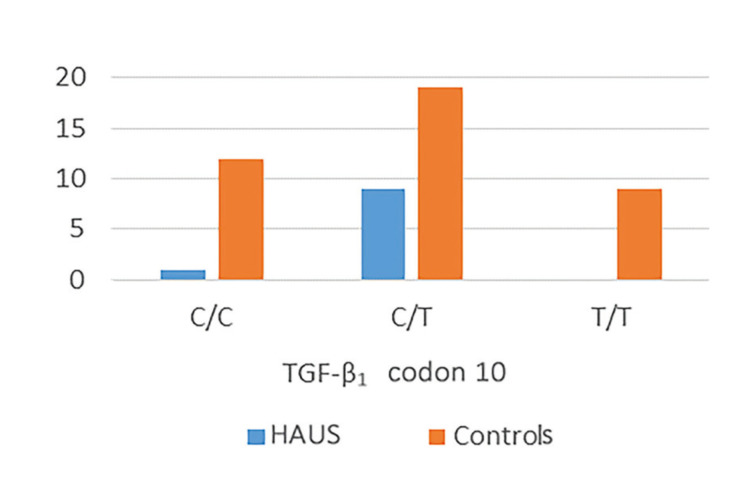
Differences in the expression of the TGF-b1 gene polymorphisms/haplotypes at codon 10 between patients with herpetiform aphthous ulcerations (HAUS) and healthy control subjects.

Similarly, in patients with with herpetiform recurrent aphthous manifestations, significant differences in genotypes distribution were recorded, compared with controls. Frequencies of C/T, C/C and T/T genotypes were determined as 90%, 10% and 0%, whereas in controls, the corresponding percentages were 48%, 30% and 22% (*p*=0.020) (Table 4 and Fig. 4).

## Discussion

Recurrent aphthous stomatitis (RAS) is one of the most frequent inflammatory disorders of the oral mucosa. Epidemiologic data indicate that the percentage of the general population affected by recurrent aphthae in different age, racial ethnic groups ranges from 5% to 20% (5). In the USA, white people are affected three times more frequently than black individuals (18). In the same country, epidemiologic studies of oral mucosa lesions in children and young adults aged 4-22 showed that about 1% of them had recurrent oral ulcers, while aproximately 35-40% reported a history of RAS-like disease, with ulcerations beginning before 5 years of age and the frequency of affcted patients increasing with age (18). Further, the highest RAS incidence in the USA was found in female student nurses (60%), male student dentists (56%) and professional school students (55%) (4). Other studies have found diverse prevalence of RAS in different populations; for example, only 0.5% of Malaysian adults were affected by RAS with an even lower prevalence 0.1% among Indians living in Malaysia (19). These findings indicate a possible role for genetic/geographic predisposition for RAS manifestation (4).

The etiology of RAS is unknown, but the immune system (including T-cells and cytokines) seems to have a crucial role via oral tolerance. Interleukin (IL)-10 is a cytokine synthesis inhibitory factor and is essential for Th2 responses. This cytokine is able to revert the immune system to rest condition after eradication of infection. Single nucleotide polymorphisms (SNPs) of IL-10 gene could alter this cytokine production (20). TGF-β is a homodimeric, 25-kDa peptide, which is essential for immunologic self tolerance as a suppressive cytokine (15). Also, TGF-β suppresses differentiation of T cells to Th1 and Th2 subsets (21). This cytokine differentiates CD4+CD25− naïve T cells to iTreg cells (CD4+CD25+ Tregs) in peripheral lymphoid organs and other tissues (22).

With regard to IL-10, several common polymorhisms have been identified within the promoter region, including -1082 G>A (rs 1800896), -819 C>T (rs 1800871) and -592 C>A (rs 1800872). According to a study by Turner *et al*. (13), allelics from mutation at locus -1082 probably affect transcription factor linkage, which is correlated with IL-10 high production *in vitro*. Therefore, IL-10 G/G genotype corresponds to IL-10 high production, whereas IL-10 A/A to low production, regardless of gene polymorphisms at loci -819 and -592. Moreover, individuals-carriers of the genotypes C/A και A/A at loci -592 express lower mRNA levels in comparison to CC genotype. In a study of common variable immunodeficiency (23), high levels of IL-10 were recorded in serum of carriers of heterozygous genotypes C/T and C/A at loci -819 and -592, respectively, in comparison to patients homozygous to allele C. It is noteworthy to mention that these three IL-10 gene polymorphisms at loci -1082, -819 and -592 are probably in linkage disequilibrium with biologic significance (23).

Regarding IL-10 gene polymorphisms in RAS, Guimarães *et al*. (24) investigated IL-10 -1082(G/A) gene polymorphisms in 64 Brazilian patients with recurrent aphthae and 64 controls, but they did not find any statistically significant difference in allelic and genotypic distribution between patients and controls. Najafi *et al*. (25) investigated frequencies of IL-10 alleles and genotypes in a group of individuals with RAS; genomic DNA of 60 Iranian patients with RAS were typed for IL-10 gene (C/A -1082, C/T -819, and C/A -592), using PCR-SSP method and frequency of each allele and genotype was compared to control group. Significantly higher frequencies of the T allele at position -819 (*p*=0.006) and the A allele at position of -592 (*p*<0.001) were found in the patients of RAS group, compared to controls. IL-10 G/A genotype at position -1082 (*p*=0.007), C/A genotype at position -592 (*p*=0.001), and C/T genotype at position -819 (*p*=0.001) were significantly higher in RAS patients. The results of this study suggest that certain SNPs of IL10 gene are associated with predisposition of individuals to RAS.

In a meta-analysis (26), IL-10 -1082 G/A polymorphism was significantly associated with the risk of RAS in a dominant model (GG+AG vs AA:OR=1,49, 95%CI=1,10-2,01, *p*=0.01). A subgroup analysis revealed significant association in Asian population in allelic, heterozygote and dominant models (G vs A:OR=1,55. 95%CI=1,04-2,31, *p*=0,03, AG vs AA:OR=1,76, 95%CI=1,16-2,67, *p*=0,01; GG+AG vs AA:0R=2,04, 95%CI=1,37-3,03, *p*=0,00). Similarly, in another meta-analysis (27), high risk of RAS was found in the allele model (G vs. A: OR = 1.308, 95% CI = 1.029–1.662, *p* (OR) = 0.028), dominant model (GG/GA vs. AA: OR = 1.45, 95% CI = 1.155–1.82, *p* (OR) = 0.001) and heterozygous model (GA vs. AA: OR = 1.327, 95% CI = 1.038–1.696, *p* (OR)=0.024), while the IL-10−592 polymorphism was significantly associated with the risk of RAS for the allele model (C vs. A: OR=0.729, 95% CI=0.549–0.969, *p* (OR) = 0.029).

In our study, statistically significant differences were recorded in genotypes distribution of IL-10 (A/C) and IL-10 (C/T) at locations -592 and -819, respectively. Frequencies of heterozygous genotypes A/C and C/T were significantly increased compared to controls. These results suggest that the aforementioned IL-10 gene polymorphisms could participate in RAS manifestation. Probably, the gene polymorphisms at locations -592 and -819 are in linkage disequilibrium with the polymorphism IL-10 -1082 (G/A), which is correlated with IL-10 production *in vitro*.

Regarding TGF-β1, Jing *et al*. (28), investigated gene polymorphism at locus -509 in patients with RAS and recorded significant differences in allele frequencies and genotype distribution. Specifically, significant differences were found in the genotype frequencies or allele frequencies of TGF-β1-509T/C site between RAS patients and controls. C/T genotype (OR = 1.231, 95% CI = 0.702-2.160), T/T genotype (OR = 2.482, 95% CI = 1.250-4.927), and T allele (OR = 1.465, 95% CI = 1.036-2.074) at the TGF-β1-509 site exhibited high risk. Yousefi *et al*. (29) investigated TGF-β1 gene polymorphisms at codons 10 and 25 and found that the greater risk of RAS occurs in individuals with C/T genotype at the codon 10 and C/G genotype at the codon 25, which leads to low production of TGF-β. This study also suggested that TGF-β single nucleotide polymorphisms could play a role in RAS pathogenesis. TGF-β1 gene polymorphism at codon 10 results from replacement of amino acid leucine with proline on signal peptide. These changes modifies a-helix, increases hydrophobia of the signal peptide core, influences TGF-β1 transportation into endoplasmic reticulum and its production *in vitro* with increased serum levels (30). In our study, differences in genotypes distribution of TGF-β1 (C/C, C/T, T/T) at codon 10 were noticed between RAS patients and controls. Interestingly, these differences were statistically significant for patients with minor recurrent aphthae, as well as for patients with the herpetiform variant of the disease.

Overall, the pathogenetic mechanism of mucosal destruction in RAS appears to represent a T-cell-mediated immunologic reaction under the regulation of cytokines that coordinate the quality and quantity of immune responce. Consequently, gene polymorphisms in involved cytokines seem to play a crucial role in the predisposition of RAS in different populations, based on geographic origin and genetic background. In our study, statistically significant differences in genotypes distribution were detected between Greek patients with RAS and healthy controls, supporting that, in this population, cytokine gene polymorphisms are correlated with the presence of RAS. Association with specific clinical variants suggest that these gene polymorphisms may also correlate with the clinical course of the disease. Nonetheless, several limitations of the present study should be acknowledged: Since it is possible that polymorphisms in other cytokine genes could be also correlated with increased risk of RAS, a more comprehensive assessment of the cytokine polymorphism profile of these patients would likely provide useful information. Further, the sampe size, although adequate, remains rather small. Moreover, the lack of long term follow up data precluded correlations with clinical parameters, other than disease type, such as symptom severity, responsiveness to treatment and recurrence rate. Therefore, the present findings need confirmation in larger patients’ cohorts with long follow-up and should be expanded to other cytokines as well.

Understanding the pattern of cytokine gene polymorphisms and the relevant molecular mechanisms could contribute to targeted treatment for RAS. The ultimate aim is the planning of targeted and individualized biological management for resistant cases in order to minimize the frequency (recurrence rate), duration and severity of the attacks.
